# Large Vessel Occlusion Stroke Secondary to Acute Aortic Dissection

**DOI:** 10.7759/cureus.9278

**Published:** 2020-07-19

**Authors:** Alec J Pawlukiewicz, Drew Long, Sumeru Mehta

**Affiliations:** 1 Emergency Medicine, Brooke Army Medical Center, Fort Sam Houston, USA; 2 Emergency Medicine, Greater San Antonio Emergency Physicians, San Antonio, USA

**Keywords:** aortic dissection, stroke, large vessel occlusion, endovascular therapy, thrombolysis, chest pain

## Abstract

Aortic dissection carries a high mortality of up to 40% at the time of initial dissection and an additional 1% per hour the dissection is untreated. Patients with acute aortic dissection most commonly present with chest or back pain. Less frequently, it manifests without pain with predominant neurologic symptoms secondary to an acute stroke. We present the case of a 53-year-old male presenting with acute onset aphasia and right-sided weakness. Incidentally, CT angiography of his neck revealed a carotid artery dissection, which was found an extension of a Stanford type A acute aortic dissection resulting in a large vessel occlusion stroke. The patient's concomitant pathologies resulted in uncertainty as to the priority of management between the interventional neurology and cardiothoracic surgery services, ultimately resulting in the transfer of the patient to an aorta specialist at an outside facility. This case highlights several areas of difficulty in the management of patients with presenting with both large vessel occlusion stroke and acute aortic dissection and the need for consideration of acute aortic dissection in patients presenting with symptoms consistent with large vessel occlusion stroke. Optimal blood pressure control is unknown, as is the ideal timing of aortic repair and the potential for endovascular therapy for large vessel occlusion stroke in the setting of acute aortic dissection. Emergency physicians must rapidly engage with neurology, interventional neurology, and cardiothoracic surgery to determine appropriate interventions and timing of operative repair. The emergency physician must consider acute aortic dissection in patients presenting with signs and symptoms concerning for large vessel occlusion stroke, even if they have no complaint of chest pain, as administration of thrombolytics in these patients may be deadly.

## Introduction

Aortic dissection (AD) is a disruption of the media layer of the aorta, typically secondary to an intimal tear, resulting in bleeding within the aortic wall and subsequent separation of the three layers of the aorta [1]. AD can be classified anatomically by the location of the intimal tear (DeBakey classification) or by whether the ascending aorta is involved (Stanford classification). AD is an uncommon but potentially deadly condition with an incidence of 2-3.5 per 100,000 person-years, with a mortality up to 40% at the time of initial dissection [1-4]. Furthermore, it carries an increasing mortality of 1% per hour the dissection is untreated, emphasizing the necessity for high suspicion and rapid detection by the emergency physician (EP). Unfortunately, AD is often difficult to diagnose on initial presentation. AD classically presents as ripping or tearing severe chest pain that is sudden in onset with radiation to the back [5,6]. Presenting less commonly as a focal neurologic deficit, acute AD may lead to an ischemic stroke through various mechanisms. We present the case of a 53-year-old male presenting to our emergency department (ED) with right-sided weakness and aphasia, found to have an extensive acute AD leading to a large vessel occlusion (LVO) ischemic stroke.

## Case presentation

**Figure 1:**
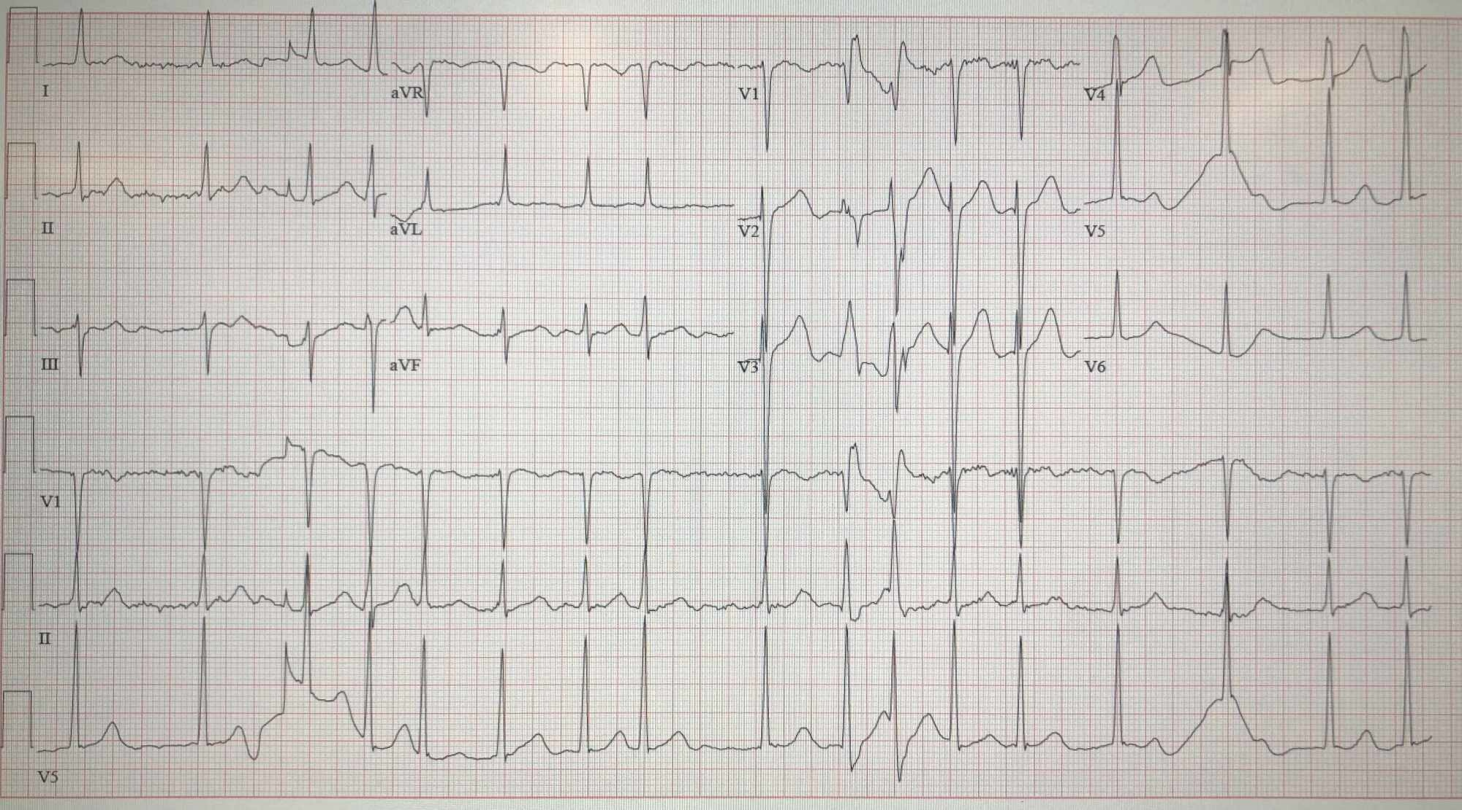
Electrocardiogram demonstrating atrial fibrillation with a rate of 83.

A 53-year-old male with a history of atrial fibrillation and an aortic aneurysm presented to our ED by Emergency Medical Services (EMS) with acute onset of right-sided weakness. Per EMS report, he was found to have right-sided weakness, aphasia, and left gaze preference approximately one hour prior to arrival. Initial vital signs upon arrival included an irregular heart rate (HR) of 99 beats per minute (bpm), blood pressure (BP) of 155/78 mmHg, a respiratory rate (RR) of 21 breaths per minute, a temperature of 97.4⁰F, and an oxygen saturation of 100%. A National Institutes of Health Stroke Scale (NIHSS) of 16 was calculated during initial evaluation with points given for inability to perform simultaneous tasks, aphasia, right upper extremity weakness, right lower extremity weakness, and gaze deviation. A stroke alert was called, and the neurology service was consulted. The patient’s electrocardiogram (ECG) showed atrial fibrillation with a rate of 83 without other significant features (Figure 1).

**Figure 2:**
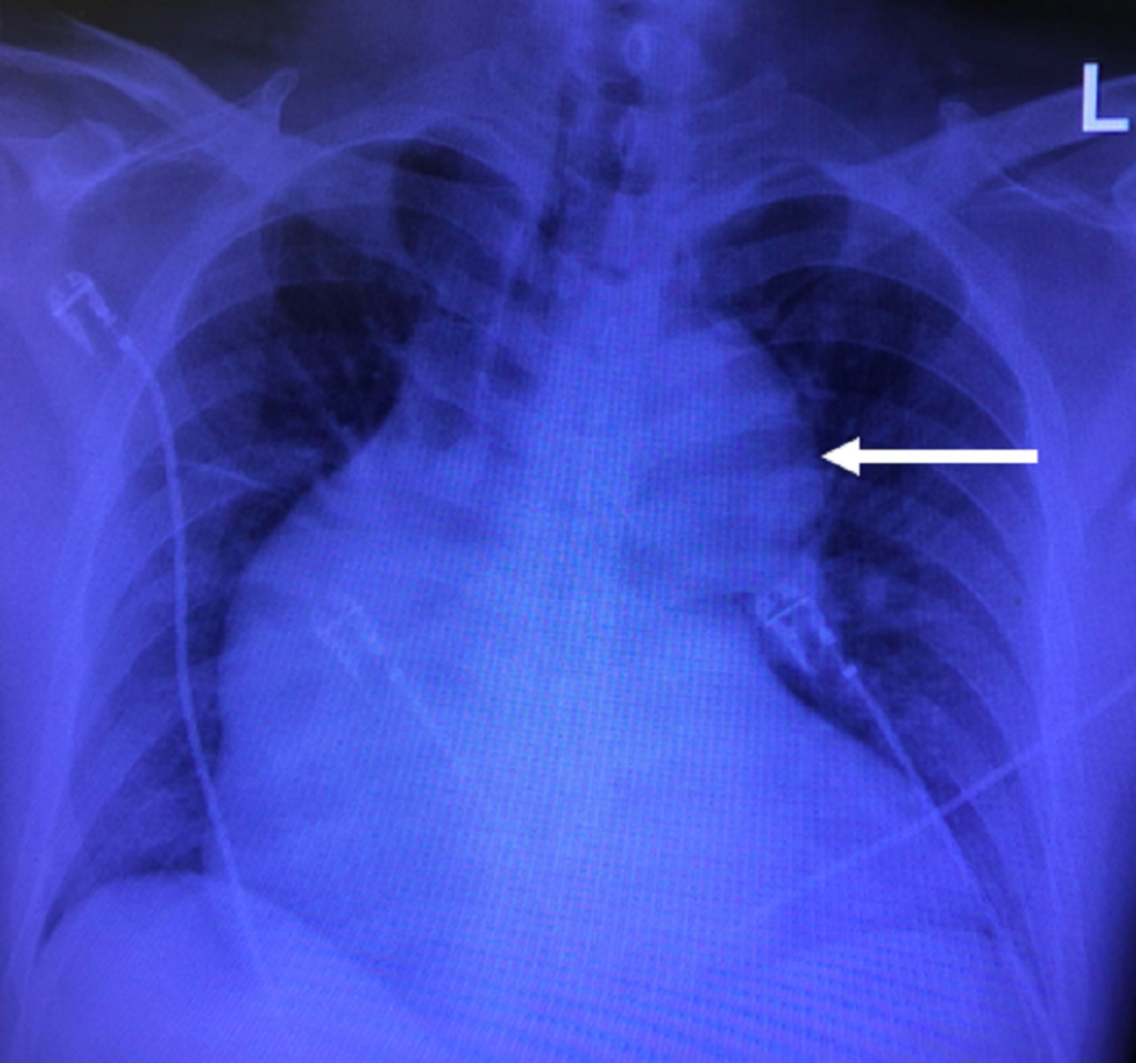
Chest x-ray with a widened mediastinum and abnormal aortic contour, indicated by the white arrow.

**Figure 3:**
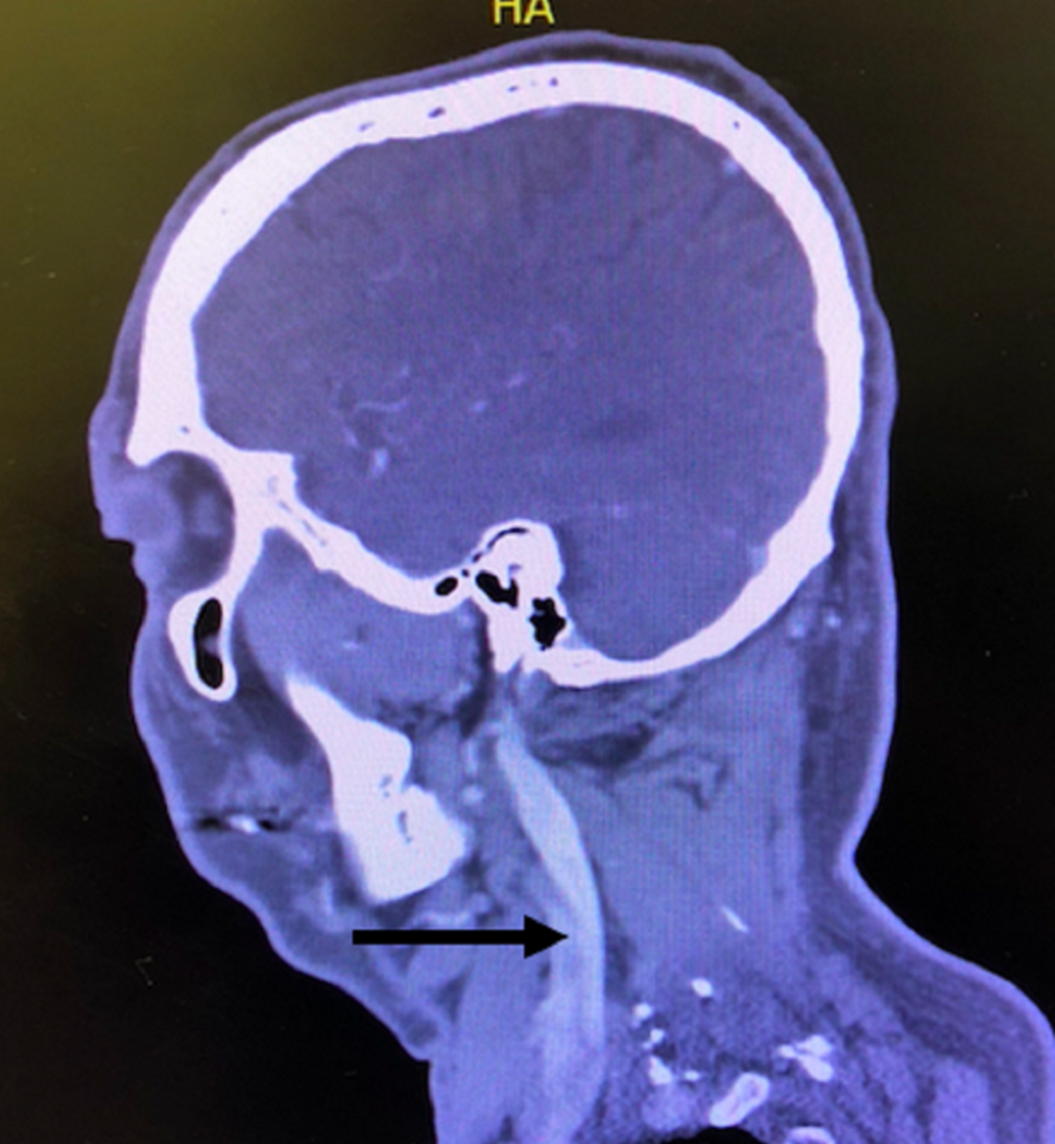
Sagittal view of CT angiography of the neck showing a left internal carotid artery dissection, with an arrow indicating the dissection.

Chest x-ray showed a widened mediastinum with enlarged aortic contour (Figure 2). CT noncontrast of the head and CT angiography (CTA) of the head and neck showed findings consistent with an early middle cerebral artery (MCA) infarct with an embolus to the M1 segment of the left MCA with extension to the proximal M2 segments, in addition to dissection of the left internal carotid artery extending into the left subclavian artery (Figure 3).

**Figure 4:**
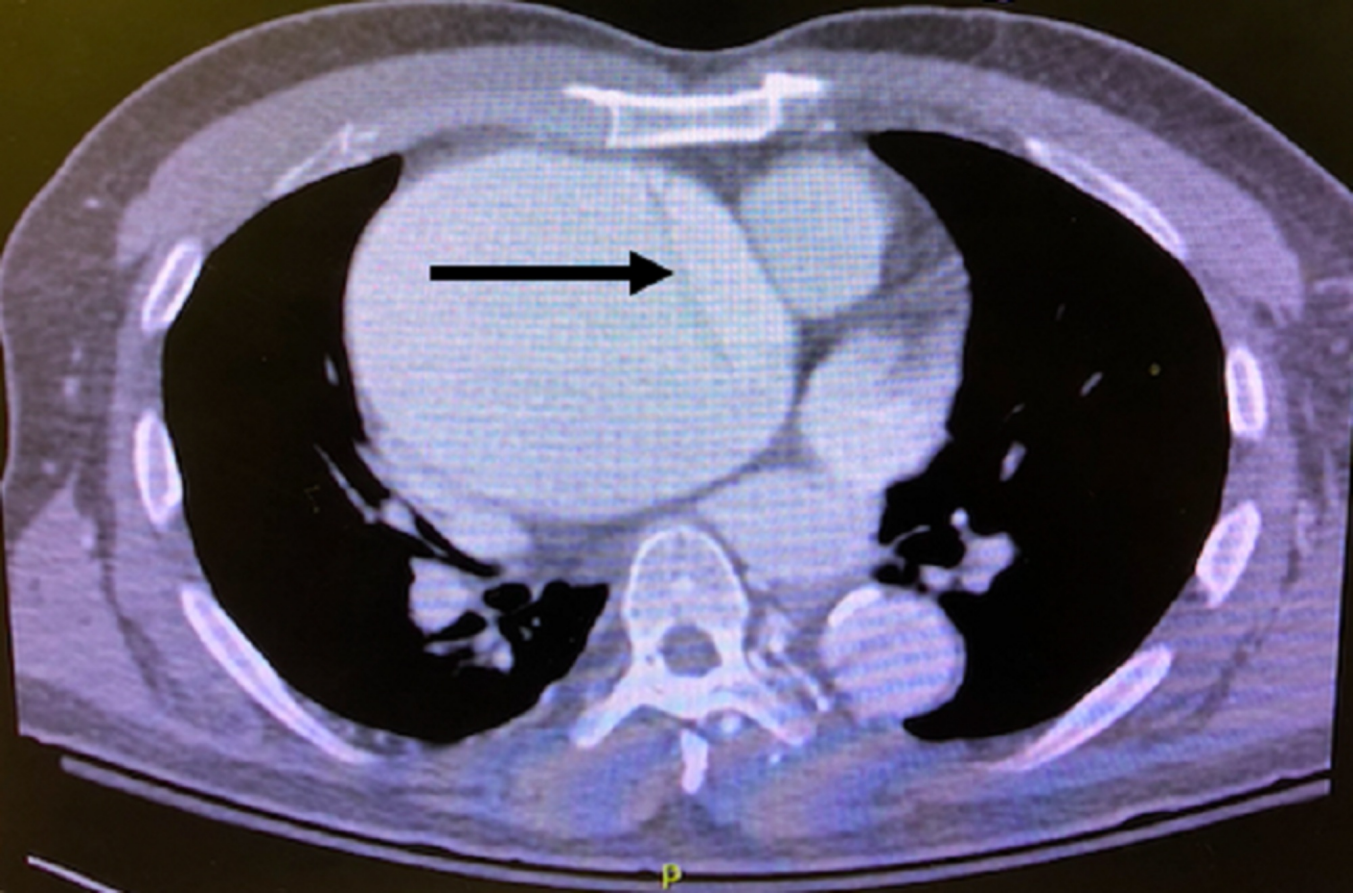
Transverse view of CT angiography of the chest showing a large aortic aneurysm with dissection, with an arrow delineating the dissection flap.

**Figure 5:**
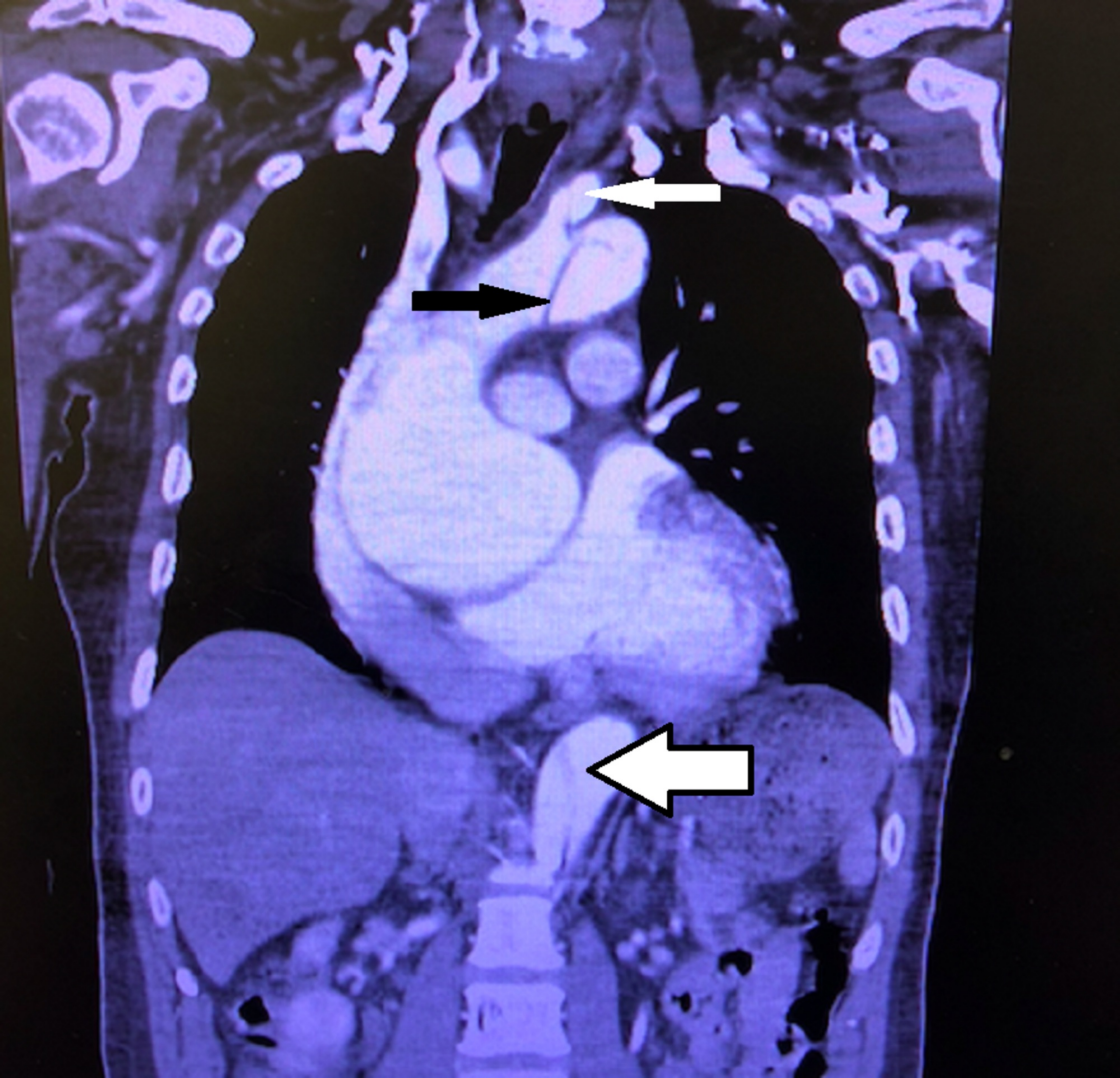
Coronal view of CT angiography of the chest showing a large aortic aneurysm with dissection, with a solid black arrow depicting the dissection in the ascending aorta, a solid white arrow depicting the dissection in the left internal carotid artery, and a white arrow with black outline showing the dissection of the descending aorta.

Given concern for an AD based on the chest X-ray and left internal carotid artery dissection, CTA of the aorta was obtained. CTA showed an ascending thoracic aneurysm with Stanford type A dissection from the aortic valve to the level of the renal arteries with extension to the left internal carotid artery (Figures 4, 5).

The patient was started on esmolol and nicardipine intravenous drips to optimize heart rate and BP with a goal heart rate of 60 bpm and goal systolic BP of 120 mmHg. The case was discussed with the neurology and cardiothoracic surgery services. Given the finding of an LVO stroke, the interventional neurology service was also consulted. The consulting physicians did not think the patient was an optimal candidate for either immediate endovascular therapy or surgical aortic repair. In conjunction with cardiothoracic surgery, the decision was made to transfer the patient to a center with an aortic specialist for operative repair. The patient was maintained on the esmolol and nicardipine infusions and in stable condition until the time of transfer. While in the ED, the patient’s neurologic exam was unchanged with persistence of his right hemiplegia, aphasia, and left gaze preference. He was ultimately transferred to a center for potential definitive care of his AD and was lost to follow-up.

## Discussion

Given the high morbidity and mortality, the EP must rapidly identify the patient with AD. Unfortunately, AD is challenging to diagnose. The most common complaints in patients with AD are chest and/or back pain, usually sudden and severe in onset [5,6]. However, patients often present atypically, and one retrospective review found 6.4% of patients presented without chest or back pain [7]. Of patients whose AD is associated with neurologic symptoms (17%-29%), approximately 53% have ischemic stroke as their causative etiology [8,9]. Patients with neurologic symptoms secondary to AD may be especially difficult to diagnose, as up to one-third of patients presenting with neurologic features do not complain of chest pain [1,9]. Initial assessment may be further complicated by aphasia, amnesia, or altered mental status, clouding an accurate history, as seen in the described case [8,9]. While neurologic symptoms may confound the diagnosis, a meta-analysis from 2018 on the history and exam features predictive of acute AD found the clinical signs most suggestive for acute AD were focal neurologic deficits, pulse deficits, and hypotension (BP < 90 mmHg) or signs of shock [10]. The EP must consider AD in patients with focal neurologic symptoms concerning for an ischemic stroke, especially if they are aphasic or altered and unable to give a history.

Optimal management of patients presenting with ischemic stroke secondary to acute AD is unknown. The co-occurrence of ischemic stroke and AD increases the possibility of complications during management for several reasons. Current emergency medicine guidelines support the administration of thrombolytics to patients presenting with acute ischemic stroke within 3 to 4.5 hours of symptom onset in patients without contraindications [11]. While there is a paucity of literature concerning thrombolytics administration in patients with ischemic stroke and acute AD, a review of the effect of thrombolytics given for myocardial infarction in patients found to have ADs showed an association with increased morbidity and mortality [12]. As giving thrombolytics to a patient with an acute AD may lead to worsening bleeding within the false lumen and progression of the dissection, acute AD is a contraindication to administration of thrombolytics.

A relatively newer therapy for management of ischemic stroke patients is endovascular therapy. Endovascular therapy has been shown to lead to improved outcomes and decreased complications in select patients with LVO strokes up to 24 hours from the onset of symptoms [13-15]. However, there are limited data on endovascular therapy for LVO stroke in the setting of AD. One case series from 2017 identified three patients with proximal right MCA occlusions in the setting of AD, all of which underwent successful recanalization with endovascular therapy and experienced improved clinical neurologic status [16]. However, two of these patients were presenting with chronic ADs that were status-post graft repair and the other had an acute type B dissection. In the presented case, neither the neurologist nor the interventional neurologist believed the patient to be an optimal candidate for acute endovascular therapy for his LVO stroke, given his concurrent Stanford type A AD. As the ultimate decision for interventional therapy does not rest with the EP, our case highlights the need to coordinate with the appropriate specialists in order to determine if the patient is a potential candidate for endovascular therapy.

Optimizing BP in AD with acute strokes presents another management challenge. The most recent American Heart Association (AHA)/American Stroke Association (ASA) guidelines for the management of acute ADs recommend initial targets of “heart rate less than 60 bpm and a systolic blood pressure between 100 and 120 mmHg" [1]. However, the most recent AHA/ASA guidelines for BP management in acute ischemic stroke recommend avoiding hypotension and maintaining BPs less than 185/110 mmHg in patients who are candidates for either tissue plasminogen activator (tPA) or mechanical thrombectomy [17]. The specific BP goals for patients with both ischemic stroke and AD have not been well defined, and should be guided in the ED by discussion with both neurology and cardiothoracic surgery specialists.

The final question raised by these simultaneous pathologies is the order of corrective procedures. In patients with ascending ADs, as was depicted in our case, surgical repair of the AD is recommended, most commonly through thoracic endovascular repair [1]. However, there is limited literature evaluating ideal operative management in patients with simultaneous AD and ischemic stroke. A case report in 2017 described a 60-year-old male with an ischemic stroke secondary to acute AD, who successfully underwent operative aortic repair five days after presentation and survived with mild residual deficits [18]. Given the overwhelming lack of data on therapy for patients with AD leading to LVO stroke, it is vital to involve all pertinent consultants as soon as an AD leading to ischemic stroke is diagnosed. If a patient has LVO stroke secondary to AD, the interventional neurologist should also be consulted for potential endovascular therapy. Patients with concomitant LVO stroke and AD are often less than ideal surgical candidates given their coexisting pathologies and the comorbidities contributing to the development of their AD. In the presented case, neither the cardiothoracic surgeon, the neurologist, nor the interventional neurologist thought the patient was a candidate for immediate operative repair or endovascular therapy, and the patient was stabilized and transferred to a medical center with an aortic specialist for potential aortic repair.

## Conclusions

Acute AD carries a high mortality rate and is often challenging to diagnose. The EP must consider acute AD in patients presenting with neurologic symptoms secondary to ischemic stroke, especially if they are unable to give a history due to altered mental status or aphasia, as administration of thrombolytics in these patients may be deadly. This case highlights several areas of difficulty in both diagnosing and managing patients with AD leading to LVO ischemic stroke. Optimal BP control is unknown, as is the ideal timing of aortic repair and potential interventional therapy for an LVO stroke. As there are limited data on management in this patient cohort, the EP must rapidly engage the neurology, interventional neurology, and cardiothoracic surgery specialists to determine appropriate interventions and timing of operative repair.
